# Radiation-induced Hounsfield unit change correlates with dynamic CT perfusion better than 4DCT-based ventilation measures in a novel-swine model

**DOI:** 10.1038/s41598-021-92609-x

**Published:** 2021-06-23

**Authors:** Antonia E. Wuschner, Eric M. Wallat, Mattison J. Flakus, Dhanansayan Shanmuganayagam, Jennifer Meudt, Gary E. Christensen, Joseph M. Reinhardt, Jessica R. Miller, Michael J. Lawless, Andrew M. Baschnagel, John E. Bayouth

**Affiliations:** 1 University of Wisconsin-Madison, Madison, WI USA; 2 University of Iowa, Iowa City, IA USA

**Keywords:** Biomarkers, Lung cancer, Non-small-cell lung cancer, Medical imaging, Biomarkers

## Abstract

To analyze radiation induced changes in Hounsfield units and determine their correlation with changes in perfusion and ventilation. Additionally, to compare the post-RT changes in human subjects to those measured in a swine model used to quantify perfusion changes and validate their use as a preclinical model. A cohort of 5 Wisconsin Miniature Swine (WMS) were studied. Additionally, 19 human subjects were recruited as part of an IRB approved clinical trial studying functional avoidance radiation therapy for lung cancer and were treated with SBRT. Imaging (a contrast enhanced dynamic perfusion CT in the swine and 4DCT in the humans) was performed prior to and post-RT. Jacobian elasticity maps were calculated on all 4DCT images. Contours were created from the isodose lines to discretize analysis into 10 Gy dose bins. B-spline deformable image registration allowed for voxel-by-voxel comparative analysis in these contours between timepoints. The WMS underwent a research course of 60 Gy in 5 fractions delivered locally to a target in the lung using an MRI-LINAC system. In the WMS subjects, the dose-bin contours were copied onto the contralateral lung, which received < 5 Gy for comparison. Changes in HU and changes in Jacobian were analyzed in these contours. Statistically significant (p < 0.05) changes in the mean HU value post-RT compared to pre-RT were observed in both the human and WMS groups at all timepoints analyzed. The HU increased linearly with dose for both groups. Strong linear correlation was observed between the changes seen in the swine and humans (Pearson coefficient > 0.97, p < 0.05) at all timepoints. Changes seen in the swine closely modeled the changes seen in the humans at 12 months post RT (slope = 0.95). Jacobian analysis showed between 30 and 60% of voxels were damaged post-RT. Perfusion analysis in the swine showed a statistically significant (p < 0.05) reduction in contrast inside the vasculature 3 months post-RT compared to pre-RT. The increases in contrast outside the vasculature was strongly correlated (Pearson Correlation 0.88) with the reduction in HU inside the vasculature but were not correlated with the changes in Jacobians. Radiation induces changes in pulmonary anatomy at 3 months post-RT, with a strong linear correlation with dose. The change in HU seen in the non-vessel lung parenchyma suggests this metric is a potential biomarker for change in perfusion. Finally, this work suggests that the WMS swine model is a promising pre-clinical model for analyzing radiation-induced changes in humans and poses several benefits over conventional swine models.

## Introduction

Functional avoidance radiation therapy has been increasingly utilized for subjects being treated for lung cancer. To do this, accurate modeling of the normal tissue complications of thoracic radiation, local functional mapping, or dose-response modeling is needed to define treatment parameters for a given patient. Previous work has indicated that radiation pneumonitis and radiation fibrosis are the primary toxicities following thoracic radiation in lung cancer patients^1–4^. In addition, these complications can be predicted using a linear quadratic model^1–6^. In recent years, multiple studies have looked at using functional metrics to create risk assessments and have found that the inclusion of these metrics increased the predictive power of those models^1,3–5,7–10^.

Several groups have used four dimensional computed tomography (4DCT) based ventilation metrics to estimate lung function^11–14^. This methodology uses anatomical changes between breathing phases to assess how the lungs expand and contract regionally. These metrics show changes with radiation dose and are surrogates for pulmonary function. Multiple groups have used (or are currently using) these metrics in clinical trials to assess the efficacy of using these metrics for functional avoidance in treatment planning^4,5,15–17^. However, the majority of these models focus on ventilation estimates and do not account for damage due to fibrosis, inflammation, or other physiological responses that may not be represented by 4DCT ventilation measures. In particular, changes in perfusion are often excluded from these functional avoidance studies which is a crucial component to the lung’s ability to function. In a functional avoidance review, Ireland et al cited that perfusion may be more clinically relevant when performing functional avoidance than ventilation^15^. Therefore, additional metrics are needed to assess this damage. One potential imaging metric is the change in Hounsfield Units (HU) prior to and post radiation therapy (RT). There have been several studies that have investigated using tissue density changes to assess lung damage^7,14,18–25^. Additionally, several studies have correlated these changes with radiation dose delivered^7,14,18–26^.

Previous work in the CT-Ventilation space has derived lung function directly using Hounsfield unit values of the time-averaged 4DCT^27^. An alternate approach uses the Jacobian determinant of the transformation computed from image registration^28^. This method assumes that the expansion of a voxel is caused by the addition of air from ventilation and is the method used in this work. This method was also shown to yield the highest DICE similarity coefficients and voxel-wise and ROI-based Spearman correlations with ventilation maps derived from 68 Ga-aerosol PET and 3He-MRI static ventilation maps, placing first in the 2019 AAPM Grand Challenge^29^. In this work we investigate the changes in HU post-RT. We assess if correlations exist between changes in HU and dose delivered or Jacobian ventilation metrics. Additionally, we investigate changes in HU in different regions of anatomy (inside and outside vessels) and assess the relationship between changes in these different anatomical regions. This leads to further analysis of radiation induced changes in perfusion and lung injury.

A current challenge of validating imaging biomarkers is finding proper surrogates for human lung function. These surrogates allow one to investigate the physiological significance of the biomarkers in addition to testing potential remedies. The use of swine in radiotherapy research has become increasing prevalent due to the swine’s similar physiology to humans. In the past decade, extensive work has been done to validate the use of swine to model human physiology in a variety of applications^30–34^. However, previous work has used conventional swine which presents barriers in analysis due to their larger size and growth rates. In this work, we present a novel swine model, the Wisconsin Miniature-Swine (WMS) developed at The University of Wisconsin-Madison. These swine were genetically engineered to present several benefits to conventional swine models. We believe these benefits allow our novel swine model to better predict these changes post-RT than conventional swine used historically.

This work provides a dose response analysis of lung density changes and Jacobian ventilation changes in human subjects enrolled in a prospective IRB-approved clinical trial. The lung density analysis outside the vasculature is also compared to the humans to validate the swine’s response to radiation therapy such that they can be used as a preclinical model for future analysis.

## Methods

### Human patient dataset

Nineteen human subjects from a prospective trial at UW-Madison (NCT02843568) investigating the use of 4DCT-based ventilation in functional planning were used. The patient cohort included non-small cell lung cancer patients undergoing radiation therapy using stereotactic body radiation therapy (SBRT). Per trial protocol, a set of two 4DCT datasets, acquired 5 min apart, were obtained before RT and at 3, 6 and 12 months post RT for each subject. During this analysis, audio coaching was used in an effort to normalize breathing patterns across subjects and reduce experimental variance. Informed consent was obtained from all participants and a summary of patient demographics is provided In Table 1. All human clinical trial procedures were approved by the University of Wisconsin Health Sciences Institutional Review Board (IRB) to ensure compliance with federal and ethical guidelines.

**Table 1:** Summary of human patient demographics.

**Number**	19
**Sex**	
M	12
F	7
**Age**	
Mean	72
Min	59
Max	85
**Prescription**	
Rx dose = 50 Gy	17
Rx dose = 60 Gy	2
**Fractionation (fx)**	
5	17
15	2
**Diagnosis**	
Adenocarcinoma	11
Squamous cell carcinoma	7
Endometral metastases	1
**Stage**	
I	3
IA	11
IB	4
II	1

### Animal model

#### Measurement setup

All Wisconsin Miniature Swine (WMS) subjects were ventilated to a consistent tidal volume (1 L was chosen to match the average tidal volume of the human subject populations). This allowed for repeatable breathing patterns in addition to assuring a fixed breath hold which is difficult to achieve with human subjects. The subjects were sedated which eliminated their susceptibility to motion artifacts in the scans and uncertainty due to patient motion in the radiation delivery. Since we were not treating a disease in the swine, we were able to design the dose distribution such that the contralateral lung was left un-irradiated for comparative analysis. Details regarding animal care and drugs administered can be found in the supplementary material. All procedures as well as animal care practices were approved by the University of Wisconsin Institutional Animal Care and Use Committee (IACUC). The drugs and methods of anesthesia and euthanasia were approved in compliance with American Veterinary Medical Association (AVMA) guidelines for anaesthesia and euthanasia of swine. Both committees assured that all procedures were in compliance with ARRIVE guidelines.

An additional benefit of using the animal model was that we had the ability to perform scans that would not typically be given to human subjects to avoid giving excess dose. This included a dynamic contrast enhanced scan.

#### Description of animal model

The Wisconsin Miniature Swine (WMS) possess several characteristics that make them an ideal model. In general, swine are well suited for biomedical studies pertaining to the development/validation of diagnostic and therapeutic technologies because of their genetic proximity to humans and similarities in anatomy^30,32–34^. The WMS were created by selective crossbreeding of several swine breeds such that their weight, size and physiology are similar to humans and their body composition can be easily manipulated^31^. As they can be easily maintained at human size for any length of time, they will remain the same size from intervention to necropsy. In addition, we were able to select swine that had lung volumes that were within the range of those in our human subjects.

The WMS at the size used in our study had lungs that matched human adult lungs. Additionally, they were swine in their early adulthood (approximately equivalent to a human in their late 20 s or early 30 s). If we had used a conventional breed of swine as previous studies have done, the swine would have been approximately 3 months of age (in order to match the size of human lungs). Given that swine reach sexual maturity at 5 months of age, a 3-month old swine is equivalent to a pre-pubescent human child at 6–8 years of age. A conventional swine at this age has a rate of development where tissue remodeling and size changes are very rapid. The swine’s ability to heal and response to radiation damage (i.e., pathophysiology) would not mimic that of a human adult. The WMS on the other hand allowed us to more closely model the pathophysiology observed in a human adult. A 14 month old WMS is close to a human in the mid-late twenties^31^.

#### Irradiation scheme for WMS

Five WMS (14.4 ± 1.7 months old) were given a research course of 60 Gy in 5 fractions approved by the an Institutional Animal Care and Use Committee (IACUC). These fractions were delivered following a standard clinical SBRT schedule receiving fractions with a day in between each delivery during weekdays and 2 days over the weekend. We chose to irradiate to 60 Gy in order to maximize the probability of inducing measurable radiation-induced toxicities. The PTV was designated as the bifurcation of a vessel in one of the lungs. Treatment delivery was done using an MRI-guided LINAC system.

#### CT acquisition

All human and swine CT images were acquired on a Siemens SOMATOM Definition Edge CT scanner. Each swine underwent six total imaging sessions (one session before each of the five fraction deliveries and one 3 months post-RT). In each session, a contrast enhanced dynamic 4DCT image was obtained. Details regarding the dynamic 4DCT procedure can be found in the supplementary material.

#### Subtracting vasculature from the dynamic 4DCTs

**Figure 1:**
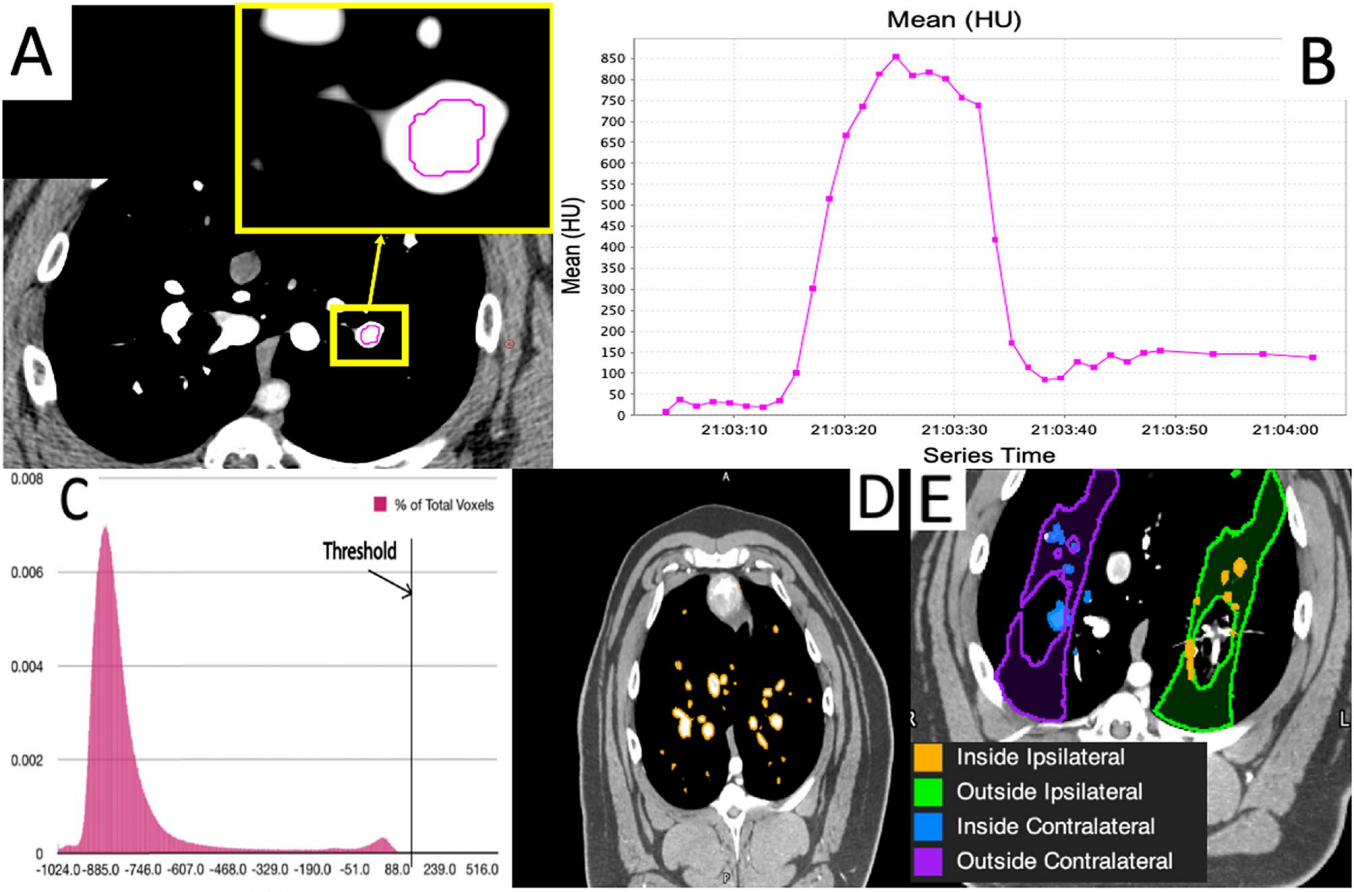
(**A**) Placement of an ROI in a region showing high contrast uptake. (**B**) Plot of the mean HU value in that ROI over the timespan of the dynamic 4DCT scan for a subject. The increase in mean HU shows that contrast is flowing in and out of this vessel. (**C**) Histogram of HU values over the entire lung mask. A threshold was established based on the second mode and was placed 2 standard deviations from the mean of the second mode. (**D**) Example scan slice showing vasculature segmented using the threshold. (**E**) Example scan slice showing the four different contours created for each dose bin to perform analysis.

Vasculature was masked out prior to analysis in the WMS swine to enable the analysis of the non-vessel lung parenchyma and the vessels separately. We used the HU values in the dynamic 4DCTs to indicate regions where contrast was present. These regions were segmented as vessels based on the assumption that the presence of contrast indicates a location through which blood is flowing. The dynamic 4DCT images contain between 28 and 36 frames. In the initial frames, contrast was not present as acquisition began before contrast was injected. After the acquisition of 4 frames, contrast injection began. As acquisitions continued after injection, the mean HU value in a vessel increased as contrast flowed in and decreased as contrast flowed out (see Fig. 1).

Segmentation was performed on a maximum intensity projection (MIP) image of all frames to account for the timing offset of contrast flowing through different vessels. All frames were deformably registered to the first frame using the built in registration algorithm in MIM Software (Cleveland, OH) prior to MIP generation.

Next, a HU threshold was utilized to perform vessel segmentation. To determine this threshold, a histogram of all pixel values over the lung mask in the first frame of the dynamic 4DCT (a frame where contrast was not present) was plotted. This histogram yielded a bimodal plot as shown in Fig. 1C. The threshold was set at 2 standard deviations above the mean of the second mode. This was done such that vessels would only be segmented if they experienced an increase in HU due to the presence of iodine contrast flow and not due to other structures in the lung that appear with a moderately elevated HU value. The standard deviation was computed using the full width at half maximum of the higher mode peak using the relation below:

1 $$\begin{aligned} FWHM=2.35 \sigma \end{aligned}$$

Finally, to segment the vasculature, a threshold was applied such that any voxel within the lung mask having a HU above the threshold was segmented. An example scan showing the segmented vasculature is shown in Fig. 1D. Once the vasculature was segmented, another contour with this vasculature subtracted was created to analyze regions outside the vasculature as well.

#### Measuring change in HU value in the swine

All post-RT scans were deformably registered to the pre-RT scan using a B-spline registration algorithm to allow for voxel-wise comparisons^35,36^. Analysis was performed in 10 Gy dose bins. This was done by importing the planned dose distributions for each patient into MIM and creating contours from the isodose lines. The dose distribution was registered to CT scan used for treatment planning. All dose contours were copied onto the contralateral lung as shown in Fig. 3C. Figure 1E shows an example of the 4 contours analyzed for each dose bin. The choice to use 10 Gy dose bins was determined through analysis of the variation in measurement as a function of the volume of the contour analyzed. As the volume of the contour analyzed decreases, the measurement becomes increasingly noisy. In SBRT treatments, high dose gradients are present thus the volume receiving a given dose is small. Our analysis found that for volumes greater than 30 cc we were able to minimize the standard deviation to below 25 $\%$ at all dose levels. This resulted in the bins being 10 Gy wide.

**Figure 2:**
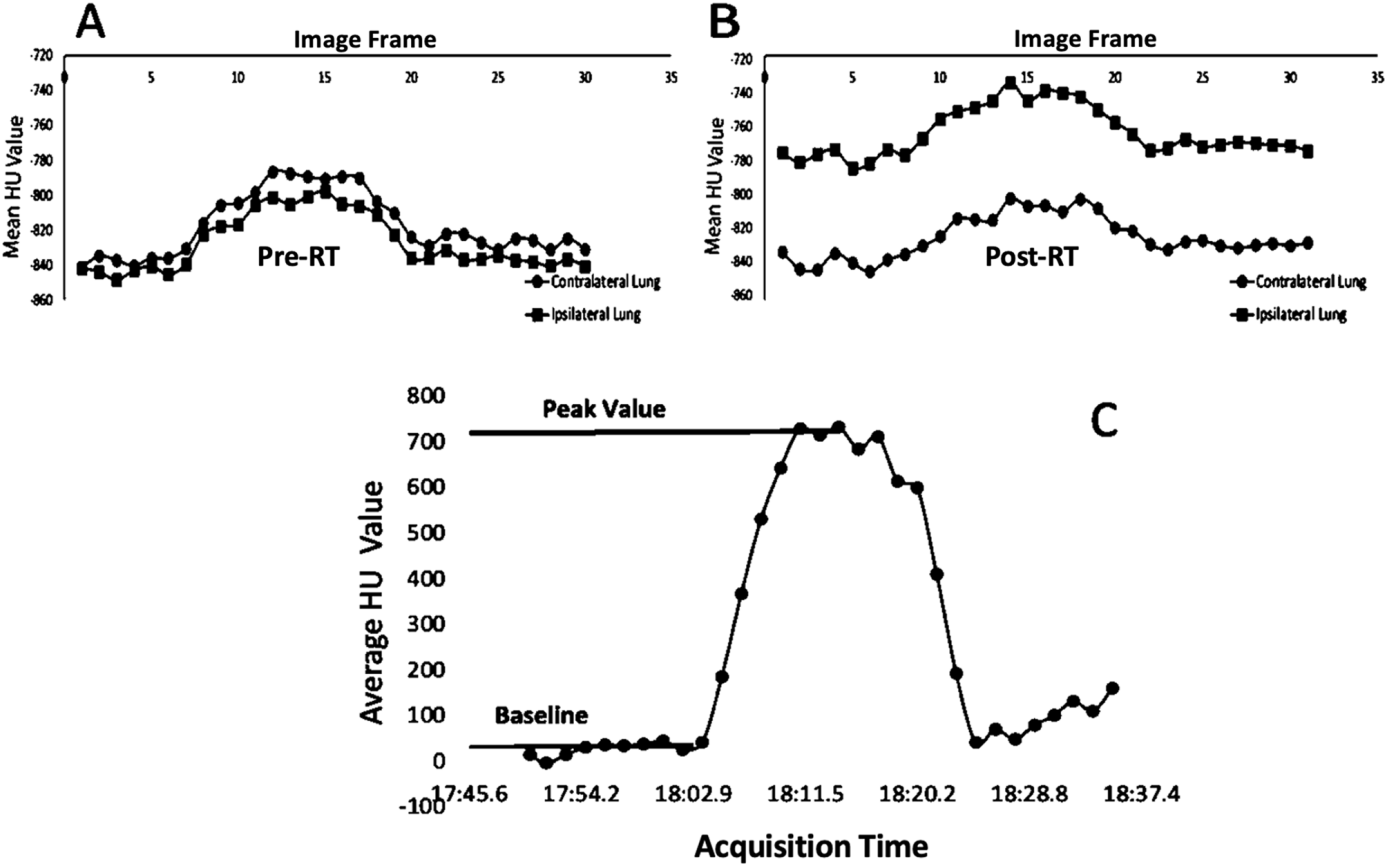
(**A**) Plot of the mean HU value of the lung parenchyma pre-RT. (**B**) Plot of the mean HU value of the lung parenchyma 3 months post-RT in a typical WMS swine and Each data point represents the average HU value in the lung parenchyma in a single acquisition (image frame) over the time trace of the dynamic contrast-enhanced 4DCT. (**C**) Plot of the mean HU value of the vessels showing the two measurements obtained. The two measurements are the baseline HU value (HU value before contrast injection) and the peak HU value (the HU value at the timepoint in the 4DCT where the contrast concentration was at it’s maximum value inside the vessels.

Figure 2 shows the two measurements that were obtained for this analysis. The mean HU value trace for the entire lung mask excluding the vasculature for one swine subject is plotted. Observe in Fig. 2B that the initial baseline values prior to contrast injection increase post-RT from the pre-RT value shown in Fig. 2A in the ipsilateral lung, but remain unchanged in the contralateral unirradiated lung.

The baseline measurement was the first measurement taken. For all swine, the first frame of the Dynamic 4DCTs was used to obtain this value in both the contours where vessels were subtracted as well as the contours of just the segmented vessels for each dose bin (following the procedures above). The second measurement was the max HU value of the trace which was taken only for the contours of the segmented vessels for each dose bin. These measurements were recorded at pre and 3 months post-RT. The percent difference was calculated using:

2 $$\begin{aligned} \Delta HU(\%) = \frac{HU_{post}-HU_{pre}}{HU_{pre}}\times 100 \% \end{aligned}$$

### Measuring change in HU value in the human subjects

The human subject analysis was similar to that of the swine with a few exceptions. Since the treatment planning was not designed to reduce radiation dose below 5 Gy in the contralateral lung, it was unable to be used as an “unirradiated” control. Additionally, since the human subjects did not receive a contrast enhanced scan, vessel subtraction was not performed and only measurements of the HU in regions of tissue that were delivered a given dose were obtained. The HU analysis was performed on the exhalation phase of the 4DCT data sets. This phase was chosen because it was believed to be the phase least susceptible to CT artifacts. Since the human subjects received two 4DCTs at the same timepoint, we computed the average of the results of the mean HU values found in each dose contour between the two scans.

### Assessing correlation between the WMS and humans

In an effort to account for differences in fractionation scheme, all plots and linear regressions were created using equivalent dose in 2 Gy fractions (EQD2) as calculated below where D is the total prescribed dose and d is the dose per fraction. An alpha/beta ratio of 3 was used for lung tissue^37^.

3 $$\begin{aligned} EQD2 = D\times \frac{d+\frac{\alpha }{\beta }}{2+\frac{\alpha }{\beta }} \end{aligned}$$

**Figure 3:**
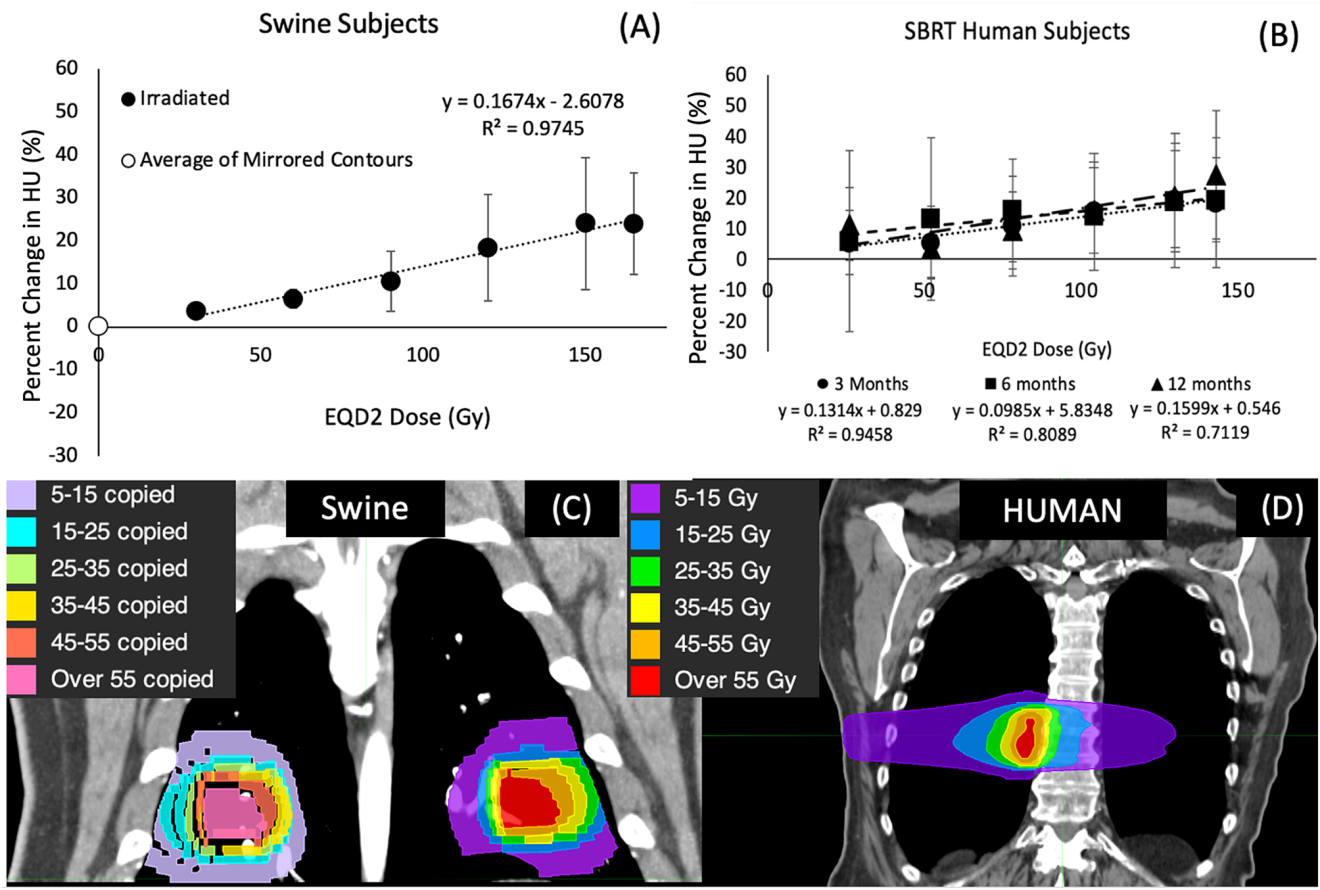
(**A**) Percent changes in mean HU post-RT in the swine. (**B**) Percent changes in mean HU post-RT in the humans. (**C**) Irradiated dose contours and copied contralateral contours in the WMS swine. (**D**) Dose distributions and contours for a typical human subject. Data is shown at 3, 6 and 12 months post RT in the humans and at 3 months for the swine. Copied contours in unirradiated regions were averaged and plotted as the 0 Gy data point in (**A**). Linear fits with R$^{2}$ values are shown for all data sets. The percent increases in all subjects were linear with strong correlations. Additionally, the unirradiated regions of the swine showed no change post-RT.

As shown in the dose distributions for an example patient in both the swine and humans in Fig. 3C,D, the volume of tissue irradiated was similar in both subject pools. However, while both subject pools received SBRT-type plans and have similar volumes irradiated, the human and swine populations received slightly different fractionation schemes (50 Gy in 5 fx in the swine vs. 60 Gy in 5 fx in the humans). When calculating correlation coefficients to compare the swine and human data, an adjusted data set was created such that there were data points for the same EQD2 values in each human population. This was done using the linear regression line of best fit equation on the swine data to calculate the percent changes in lung density at the EQD2 values recorded in the human subjects. Since the linear regression fit was so strongly correlated (R^2^ = 0.987) we expect the error resulting from doing this to be negligible. These adjusted data sets were used to calculate the Pearson correlation coefficients for the swine and the human subjects.

### Ventilation change measurement via Jacobian analysis

4DCT can be used to compute a surrogate for regional ventilation of lung tissue and provide a spatial map of the local lung tissue expansion and contraction using Jacobian analysis^6^. In this work, Jacobian analysis was used as a surrogate to assess change in ventilation in the human subjects. The Jacobian values were calculated on all images following the methodology described in Patton et al.^6^. A Jacobian value equal to one represents no local volume change, greater than one represents expansion and less than one represents contraction of the lung tissue. Equivalent tidal volumes (ETV) were used to select the phases of the 4DCTs used for the inspiration and expiration scans. This has been shown to increase the repeatability of the ventilation measurement and isolate the effect of radiation in longitudinal changes^38^.

To assess the relative change in ventilation at different timpoints post-RT, the Jacobian ratio was calculated using the relation:

4 $$\begin{aligned} Ratio = \frac{J_{post}}{J_{pre}} \end{aligned}$$

A Jacobian ratio of 1 indicates the voxel did not change from its pre-RT elasticity, a ratio of less than one indicates the voxel was less elastic post-RT compared to pre-RT (loss of ventilation) and a ratio of greater than one indicates a more elastic voxel (improved ventilation) post-RT. For this work, a threshold of a 0.95 Jacobian ratio was used to determine the voxels that were considered damaged post-RT. This threshold was used in the works of Patton et. al and Wallet et al and was chosen for consistency. Additionally, it is the threshold used for evaluation of damage in the clinical trial the human patients were taken from^6,39^. Dose binned analysis was performed using the same contours created for the HU analysis. In each dose bin the percent of voxels with a Jacobian ratio value of 0.95 or lower was calculated.

### Statistical analysis

Student paired two-tailed t tests were used to compare the pre and post-RT HU values. A Kolmogorov–Smirnov test was used to verify normality^40^. Correlations between the WMS and human subjects as well as all percent increases with dose were assessed using Pearson correlation coefficients. Additionally, all linear fits to data were evaluated using the coefficient of determination, R^2^. To correct for multiple comparisons, Bonferroni adjustment was used. This method of adjustment is the most conservative of adjustments and yielded an adjusted p value threshold of 0.01 for statistical significance at $\alpha$ = 0.05 level. All results were still statistically significant under these adjusted criteria^41^.

## Results

### Observations of HU change

#### Swine subjects

Statistically significant increases in the mean HU value were seen in each dose bin of the swine in the non-vessel lung parenchyma. The percent increases showed a linear dependence with the EQD2 dose delivered with Pearson coefficient of 0.989 (p = 0.001). The linear regression fit had a slope of 0.167 %/Gy. In the contours copied onto the unirradiated lung, there was no statistically significant changes seen in the mean HU values. The percent increases of all contours are shown in Fig. 3A.

**Figure 4:**
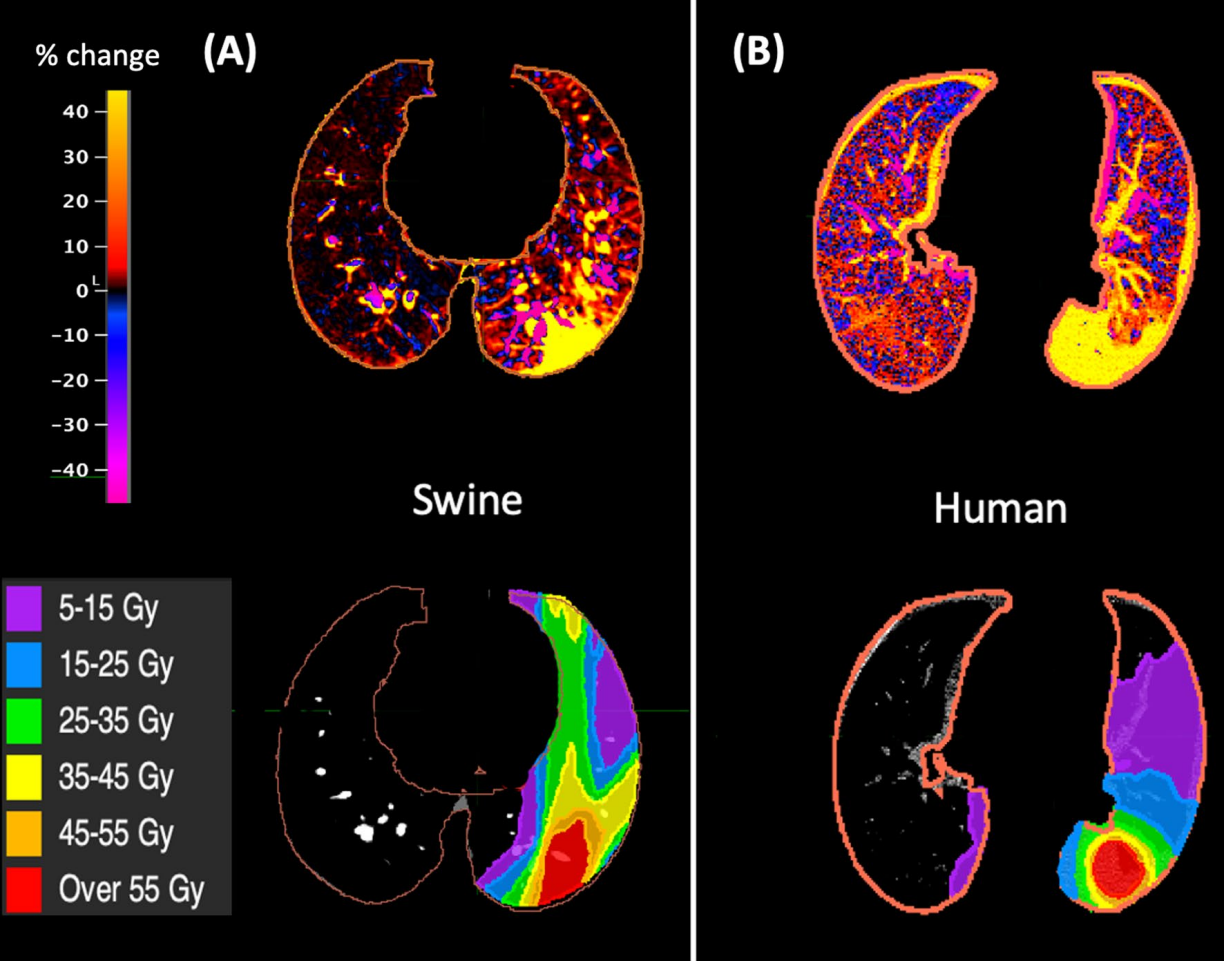
(**A**) Heat map showing the percent change in HU in each voxel with the corresponding dose distribution for a typical WMS swine (**B**) for an SBRT treated human. In all cases, large increases in HU are localized to areas receiving radiation.

The heat map shown in Fig. 4A shows the percent change in HU for each voxel of the post-RT scan as compared to the pre-RT scan. Below the heat map is the dose distribution that was delivered to the subject. It can be seen that the large increases in HU are localized to irradiated regions.

**Figure 5:**
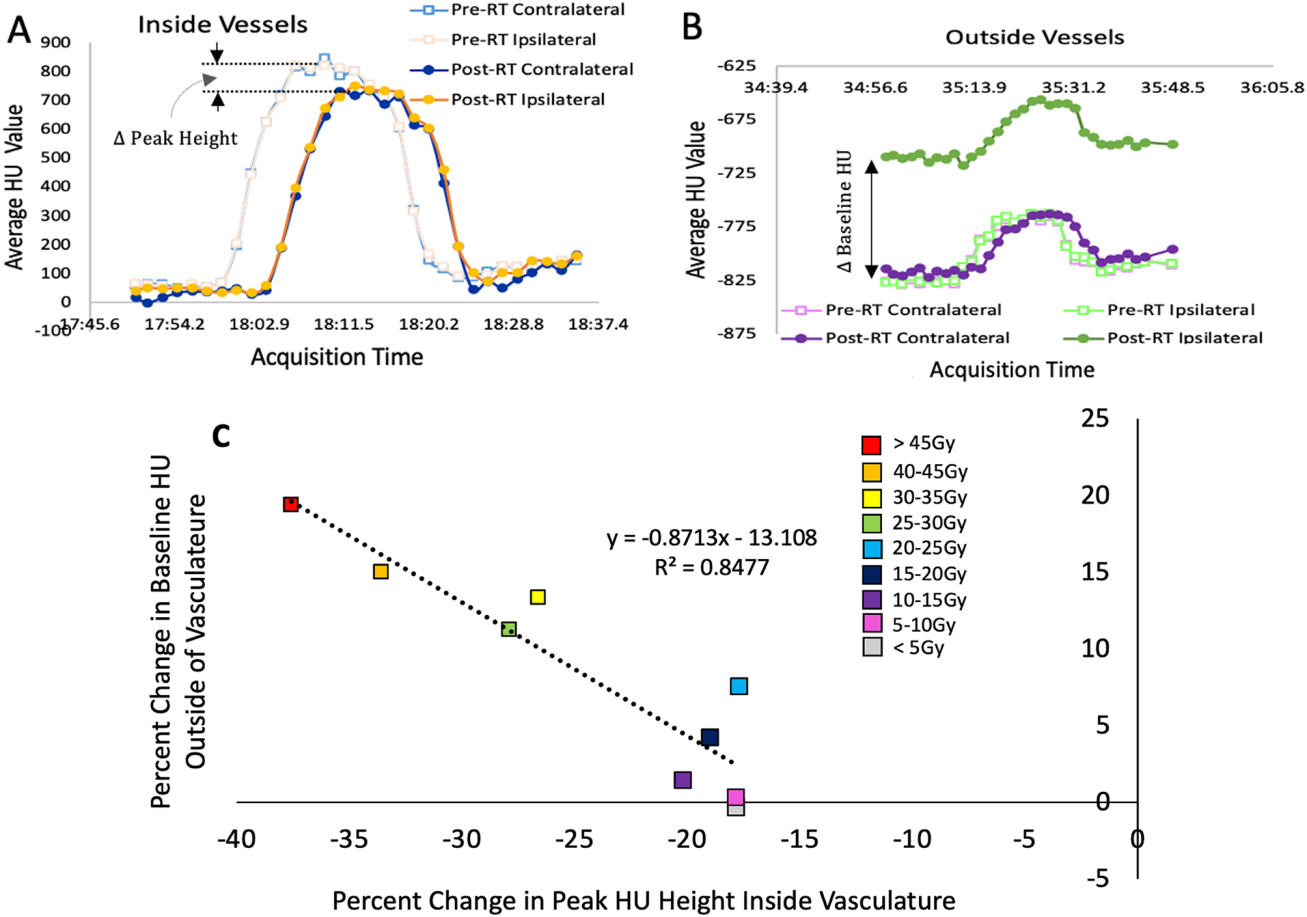
(**A**) Example contrast flow in the vessels pre- and post-RT for one WMS subject in the 20 Gy dose bin; by comparison the post-RT peak HU is reduced in magnitude. Here the acquisition times shown are the time of day for the pre-RT data. Since the specific times are insignificant, the post-RT data is plotted on the same timepoints. The important feature is the difference in the pre and post-RT peak HU values. (**B**) Example contrast flow outside of the vessels for the same WMS subject in the 20Gy dose bin; the post-RT ipsilateral region shows increased HU outside the vessels while the contralateral region does not increase. (**C**) Reduction in HU inside the vessels corresponds to an increase outside the vessels. Each point on the line corresponds to a different dose bin analyzed and is the average of all WMS subjects.

Additionally, statistically significant decreases (p < 0.05) in the peak mean HU were seen in each dose bin of the swine in the segmented vasculature. The percent changes in HU outside the vessels were strongly correlated to the percent change in peak HU inside the vessels at 3 months post RT as shown in Fig. 5.

#### SBRT human subjects

Table 1 of the supplemental material and Fig. 3B show statistically significant increases in all dose bins except 5–15 Gy (EQD2 = 13–39 Gy) for the human subjects. Table 1 lists the volume of each contour. The increases in HU were linearly correlated with dose delivered. Pearson correlation coefficients were 0.973, 0.899, and 0.844 for 3, 6 and 12-months post-RT, respectively, with p values all less than 0.00001.

The heat map shown in Fig. 4B shows the percent change in HU for each voxel of the 3 months post-RT scan compared to the pre-RT scan for a single subject. Below the heat map is the dose distribution that was delivered to the subject. Notice that the large increases in HU are localized to irradiated regions.

#### Correlations between swine and humans

**Figure 6:**
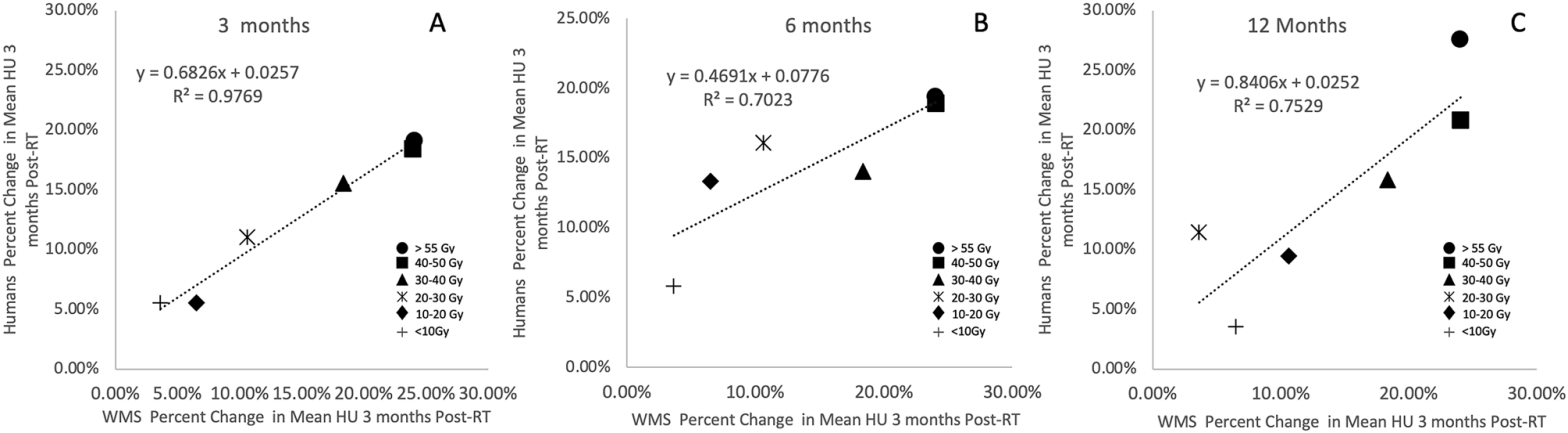
Correlation between the changes seen in the swine at 3 months post-RT and the humans at different timepoints post-RT. Each point represents a dose bin where analysis was performed. (**A**) Correlation between the humans at 3 months and swine at 3 months. (**B**) Correlation between the humans at 12 months and swine at 3 months. A curve was fit to all data sets and Pearson stats showed strong correlation with the swine data.

Figure 6A,B shows the post-RT percent changes in mean HU value in the human subjects plotted vs. the percent change seen in the swine for the same dose bin at 3-months post-RT. Data was not obtained beyond 3 months post-RT in the swine and the swine data plotted is the adjusted data set derived from the linear best fit equation to match the EQD2 values measured in the humans. The humans show strong linear correlation with the swine data. The Pearson coefficients for the humans were 0.973, 0.899 and 0.844 (p < 0.05) and had linear fits had slopes of 0.785, 0.588 and 0.955 % change in human per % change in swine at 3, 6 and 12 months, respectively.

### Change in Jacobian results

**Figure 7:**
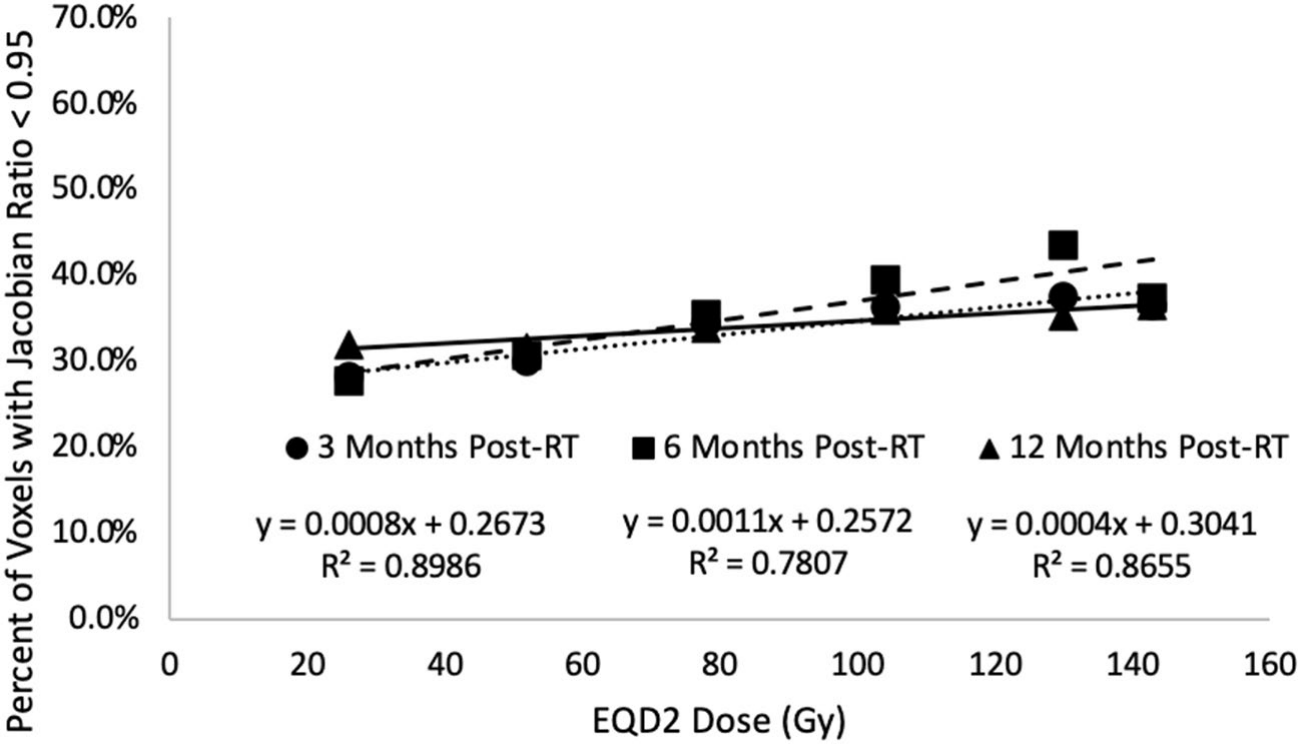
(**A**) Percent of voxels experiencing a 5 % or greater reduction in Jacobian value post-RT at each dose level in the humans. Results show a linear increase in the percent of voxels experiencing the reduction in ventilation with increasing dose. Data is shown at 3, 6 and 12 months post-RT. The number of subjects analyzed at each timepoint are 19, 13 and 11.

Figure 7 shows the percent of damaged voxels in each dose bin for the humans at 3, 6 and 12 months post-RT. The percent of damaged voxels increases as the dose delivered increases and is correlated linearly at all timepoints. Additionally of note, there was little difference between the datasets at the different timepoints in SBRT subjects.

### Correlation between change in Jacobian and change in HU

**Figure 8:**
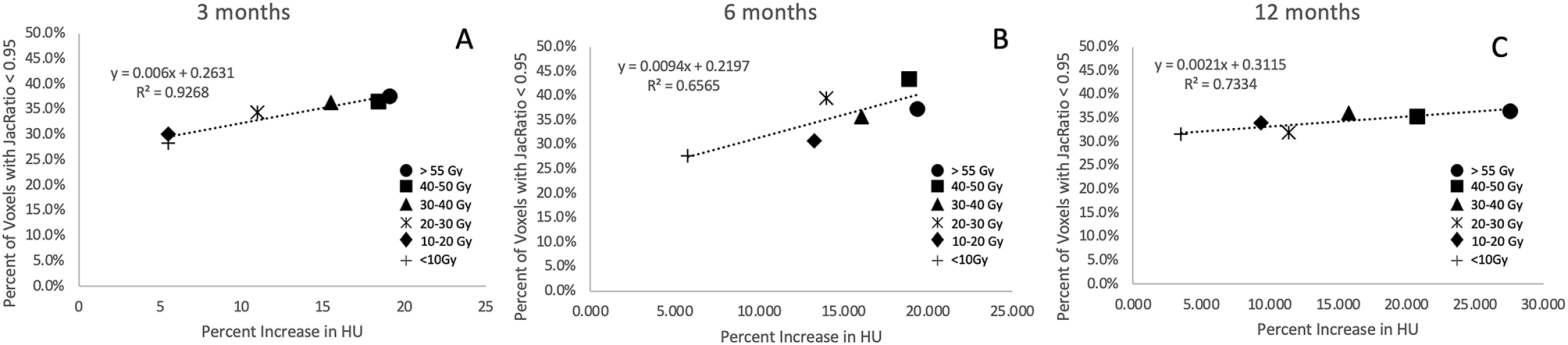
Correlation by dose bin of the percent of voxels with Jacobian ratio less than 0.95 with the percent increase in HU at (**A**) 3 months post RT (**B**) 12 months post-RT. While there are strong correlation coefficients observed at 3 months, the slopes of the best fit lines are very low indicating no real relationship between the two metrics. Each point on the plots represents a dose bin analyzed.

Figure 8 shows the percent of damaged voxels plotted against the percent increase in HU observed for the humans at the 3 and 12 month timepoints. Each point on the plot represents a different dose bin analyzed. While strong correlation exists at both timepoints, the slope of the linear fit is very low indicating that the changes in HU observed are not indicative of the ventilation changes represented by the change in Jacobian.

## Discussion

### Observed radiation induced changes

#### Observed increases in HU post-RT outside vasculature

Statistically significant increases in the mean HU value were observed in both the human subject and WMS groups 3 months post-RT. Increases ranged from 3.6$\%$ in the lowest dose bin to 30.0$\%$ at the largest doses and increased linearly. In regions receiving less than 5 Gy in the swine, no statistically significant changes in HU were observed indicating changes were radiation-induced.

The percent increases in HU increased linearly with dose at all time points for the human subjects. We believe these changes model a combination of an inflammatory response that resolves with time combined with longer lasting damage that remains. The proportion of each effect’s contribution cannot be distinguished from these measurements alone. Examples of longer lasting damage are pneumonitis which begins to develop 2–4 months post-RT and fibrotic tissue which typically can develop 4–6 months post-RT^1^.

All heat maps showing the percent changes in HU indicated the changes in HU value are localized to regions receiving dose. In addition, the copied contours in unirradiated sections of lung in the swine showed no statistically significant differences post-RT. This indicates the changes we are measuring were radiation-induced. It is worth noting that by using the vessel subtraction method we chose, it is possible that the voxels at the edge of the vessel were excluded from the subtraction and thus analyzed in the regions classified as “non-vessel lung parenchyma”. This is due to the increased HU inside the vessel from contrast being present setting the threshold for vessel classification higher. However, these voxels are not a significant contribution to our analysis when averaged with all the additional voxels in the contour. This is proven in the contralateral lung contours where vessels were subtracted in the same manner and yet we saw less than a 0.5$\%$ increase in HU.

#### Correlation between HU changes in and out of vasculature

A full understanding of the physiological processes the change in HU represents has yet to be developed. From our results looking at the change in HU inside and outside of vessels, we speculate that part of the physiological response modeled by these changes in HU is a radiation-induced loss of perfusion. Radiation-induced changes in perfusion have been shown by other groups via PET and dual-energy CT (DECT) methods^8,10,42,43^. Additionally, functional avoidance trials have shown there is a benefit to avoiding highly perfusing regions^26^. The correlation of the increases in HU outside the vasculature combined with the decrease in mean peak HU in the regions of vasculature suggest that some of the HU increase is due to radiation induced vasculature leakage of contrast. While this does not directly confirm change in perfusion, a change that would have to be seen at the capillary level, our hypothesis is that this can be inferred from these results since the reduction in HU inside the vasculature would indicate a loss of contrast (a high HU material) and the corresponding raise in HU outside these vessels would explain where the contrast in addition to blood (another higher HU material) is going.

The reduction in contrast inside the vasculature was systemic and occurred in both irradiated and non-irradiated regions. However, the increases post-RT were only in the irradiated regions. This supports our hypothesis since leakage of contrast would result in less contrast circulating through all vasculature (showing a reduction in peak HU height), but the increase in HU due to contrast leakage would only be seen where the source of the leak is (in the irradiated lung tissue). Additionally, this result was observed across all swine subjects. When analyzing the plot in 5c, it appears as if there is a threshold dose above which this leakage occurs of 25 Gy. All dose bins plotted that are greater than 25 Gy showed greater than a 10% increase in HU outside the vasculature while dose bins that were less than 25 Gy showed below 10% increases outside the vasculature. The slope of curve 5c is − 0.87 which is is dominant but not a perfect negative relationship, suggesting additional secondary factor(s) contributing to the increase in HU outside the vasculature. encompasses this change in perfusion as well as acute damage. As mentioned previously, there are other mechanisms of damage such as inflammation and presence of fibrotic tissue that would also contribute towards a raised HU value. Future work will involve acquiring these perfusion scans on human subjects receiving SBRT, in addition to analyzing damage to the pulmonary vasculature through post-RT lung pathology of the swine to confirm this speculation. If verified the change in HU may be a potential bio-marker for radiation-induced change in perfusion and understanding the dose threshold above which this damage occurs would provide additional guidance during planning.

#### Interpretation of Jacobian results

Figure 7 indicates that the human subjects showed no difference in the percent of damaged voxels (as indicated by a 0.95 or lower Jacobian ratio) across the 3 timepoints measured. Additionally, the percent of damaged voxels (those seeing a 5$\%$ or greater reduction in Jacobian) was independent of dose delivered above an EQD2 of 20 Gy.

One important point that should be considered is that these results encompass subjects pulled from a clinical trial studying the effectiveness of functional avoidance radiation therapy where highly ventilating regions were avoided. The results are not stratified by subjects in the control vs the experimental arm. It is possible the subjects in the experimental arm may have experienced different changes in Jacobian post-RT than those in the control arm since the tissue that was irradiated was selectively optimized to irradiate more low-functioning tissue.

As indicated in Fig. 8, the percent of voxels showing a decrease in Jacobian were not correlated with the changes in HU observed with the exception of the 3 months timepoint. Additionally, while a significant percentage of voxels (greater than 30%) were damaged, the dose delivered did not heavily influence the number of voxels damaged as previously described and shown Fig. 7. However, the dose delivered did heavily influence the changes in HU. These results indicate that while radiation does induce changes in ventilation (which has been seen by several other groups as well)^4,5,13^, there are additional radiation induced changes taking place that are not indicated by the Jacobian metric and these changes are highly influenced by the magnitude of dose delivered.

This observation is important as there are several groups using the difference in HU between inhale and exhale images to estimate ventilation^44–46^. Therefore it would be reasonable to speculate that the changes in HU we see in this work are correlated with and partially caused by a change in ventilation. However, our results looking at Jacobian-based ventilation estimates show no correlation with change in HU. This suggests that the cause for the change in HU involves mechanisms other than ventilation. Combined with our contrast data and results discussed previously, this work suggests that, in addition to some component of fibrotic damage, these increases in HU are representing leaked blood from vasculature and ultimately perfusion change.

### Correlations between animal model and humans

Data was not obtained beyond 3 months post-RT in the WMS model. However, the human data sets showed a strong linear correlation at all timepoints with the WMS data. Additionally, the linear fit for the 12 month post-RT SBRT human data vs Swine 3 month data showed a slope of 0.954 indicating that the swine results closely modeled the human results at 12 months. The linear fit for the 3 month data in the SBRT humans vs the 3 month swine data had a slope that was less than this (0.704). This indicates that the swine show an accelerated response to the humans. This accelerated response in the WMS model is consistent with reports in literature of swine models experiencing an expedited biological response when compared to humans^30,32–34^. This feature poses as a potential benefit to using the swine for pre-clinical modeling as it provides the ability to see response quicker in the animal model which could provide insight for human study design.

The 6 month data did not correlate as strongly with the swine data. We believe this is because at this timepoint there are multiple mechanisms present that are causing change as described in “Observed increases in HU post-RT outside vasculature”. Some of these changes, such as inflammation, are early effects that would be beginning to resolve at 6 months, while others are late effects such as fibrosis which could be starting to present themselves at 6 months in the swine. The combination of these effects is a plausible cause of the lower correlation at this timepoint.

### Clinical impact

This work quantifies an anatomical response to radiation dose in a human population as well as in an animal model. Our results in the human population looking at changes in HU are in agreement with the behaviors reported in literature^20,23^. Additionally our results were part of a prospective clinical trial designed to standardize the acquisition of images for all subjects and also analyzed data longitudinally over multiple timepoints. We speculate that the observed increases in HU are indicative of multiple mechanisms consisting of a combination of early inflammatory effects as well as late toxicities. Other groups have demonstrated radiation-induced changes in perfusion using SPECT^43^. Our findings in the swine indicate that there may be the ability to infer these changes from 4DCT which would immensely aid translation to a clinical setting since 4DCTs are already routinely collected for treatment planning and would not require the acquisition of additional scans. These results would also present an opportunity for superior functional avoidance therapy as other groups have suggested that perfusion may be more clinically relevant when performing functional avoidance than ventilation^15^. However, further investigation is required to verify and validate this hypothesis and future work will involve attempting to confirm these speculations using pathology studies.

Correlations of the swine and human subjects shows the potential to use the swine as a pre-clinical model for human response. This work shows strong correlation with human outcomes and additionally shows accelerated response. This presents a benefit in being able to expedite the process of assessing response in the swine lungs in order to better design human clinical trials and investigation of potential treatments and intervention mechanisms. As mentioned previously, the novel swine used in this study present multiple benefits over conventional swine. These benefits combined with the results of this study presents these animals as an improved option for more longitudinal studies due to the swine’s ability to stay the same size and mature similarly to an adult human.

After the 3-month post-RT scan, the swine lungs were extracted from the animal for future pathology studies. This work will provide further insight regarding the physiological response of these subjects and the damage done to the vasculature. Future work will also include a clinical trial analyzing the response of a larger sample size of these novel swine as well as an analysis of contrast enhanced scanning on humans. This will enable faster development of predictive models that would be able to be validated on existing human subject data from this trial. From there, clinical trials assessing the effectiveness of intervention mechanisms on human subjects may be initiated.

## Conclusion

Radiation induces changes in pulmonary anatomy post-RT. This work quantifies those changes using a HU analysis and shows that the WMS model is a good surrogate for analyzing radiation-induced changes in humans treated with SBRT. The presentation of this model as a pre-clinical model yields an opportunity to expedite the development of predictive models as well as human clinical trials to assess various response and intervention mechanisms in human subjects. The observations connecting the changes in HU to changes in contrast present a potential bio-marker for analyzing functional changes in perfusion that can be derived off of 4DCT as opposed to requiring additional scans outside of clinical protocol. This would allow these metrics to be considered in functional avoidance therapy and could provide a significant benefit to patient outcome.

## Supplementary Information

Supplementary Information 1.
